# Intrathecal Transplantation of Human Umbilical Cord‐Derived MSCs Outperforms Intravenous Administration in Attenuating Pathology and Cognitive Deficits in Alzheimer's Disease Mice

**DOI:** 10.1002/cns.71215

**Published:** 2026-11-04

**Authors:** Li Ding, Wei Yu, Yuqian Chen, Hu Wang, Shuya Li, Hanyu Li, Tianyu Shu, Xiaohe Huang, Ying Yang, Rui Liu, Xinglin Liu, Zhibing Ai, Xingrong Guo, Jun Chen, Qiumin Qu

**Affiliations:** ^1^ Department of Neurology The First Affiliated Hospital of Xi'an Jiaotong University Xi'an China; ^2^ Department of Neurology, Hubei Provincial Clinical Research Center for Umbilical Cord Blood Hematopoietic Stem Cells, Hubei Key Laboratory of Embryonic Stem Cell Research Taihe Hospital, Hubei University of Medicine Shiyan Hubei China; ^3^ Neuroregeneration and Stem Cell Programs, Department of Neurology, Institute for Cell Engineering Johns Hopkins University School of Medicine Baltimore Maryland USA; ^4^ Department of Anatomy Hubei University of Medicine Shiyan Hubei China; ^5^ Laboratory Animal Center Hubei University of Medicine Shiyan Hubei China

**Keywords:** Alzheimer's disease, cognitive deficits, human umbilical cord‐derived mesenchymal stem cells (HUCMSCs), intrathecal transplantation, intravenous administration

## Abstract

**Background:**

Mesenchymal stem cell (MSC)‐based therapies have emerged as a promising approach for Alzheimer's disease (AD), owing to their neuroprotective, anti‐inflammatory, and immunomodulatory properties. However, the optimal delivery route for maximizing therapeutic efficacy within the central nervous system remains unclear. This study aimed to directly compare the therapeutic effects of intravenous versus intrathecal transplantation of human umbilical cord‐derived mesenchymal stem cells (HUCMSCs) in a transgenic AD mouse model (5xFAD).

**Methods:**

HUCMSCs were isolated, characterized, and administered to 5xFAD mice via intravenous or intrathecal routes. Cognitive function was assessed using the Morris water maze. Amyloid plaque burden, microglial activation, microglial barrier integrity, and axonal dystrophy were evaluated through immunofluorescence and confocal microscopy. The expression of TREM2, DAP12, and Syk was analyzed to elucidate underlying molecular mechanisms.

**Results:**

Intrathecal transplantation of HUCMSCs significantly improved spatial learning and memory in 5xFAD mice compared to intravenous delivery. HUCMSC treatment substantially reduced amyloid plaque burden and axonal dystrophy, while promoting microglial activation and enhancing the protective function of the microglial barrier. Additionally, HUCMSC administration upregulated TREM2, DAP12, and Syk expression, with intrathecal delivery inducing more robust activation of these microglial signaling pathways than intravenous infusion.

**Conclusions:**

HUCMSC transplantation alleviates cognitive deficits and modulates key pathological and molecular features of AD in the 5xFAD mouse model, with intrathecal administration demonstrating superior therapeutic efficacy compared to intravenous delivery. These findings underscore the critical importance of optimizing cell delivery routes to enhance the clinical potential of stem cell‐based interventions for AD.

## Graphical abstract

Mesenchymal stem cell therapy is a promising approach for Alzheimer's disease, yet the optimal delivery route remains unclear. Using the 5xFAD mouse model, we performed a head‐to‐head comparison of intrathecal and intravenous transplantation of human umbilical cord–derived mesenchymal stem cells. Intrathecal delivery more effectively improved cognition, reduced amyloid pathology, and enhanced microglial barrier integrity, highlighting delivery route as a critical determinant of efficacy.

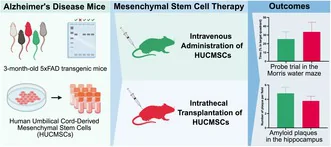

## 1 Introduction

Alzheimer's disease (AD) is a devastating neurodegenerative disorder characterized by progressive cognitive decline, synaptic dysfunction, and characteristic neuropathological hallmarks, including extracellular amyloid‐β (Aβ) plaques and intracellular neurofibrillary tangles [1, 2]. As the most common cause of dementia, AD currently affects millions of individuals worldwide, with its prevalence expected to rise dramatically due to global population aging [3]. Despite decades of research, the development of effective disease‐modifying therapies remains a critical unmet need, underscoring the urgency of identifying novel strategies capable of halting or reversing neurodegeneration [4]. Among emerging therapeutic approaches, stem cell‐based therapies—particularly mesenchymal stem cell (MSC) transplantation—have attracted considerable interest due to their neuroprotective, anti‐inflammatory, and regenerative properties, positioning them as a promising avenue for AD intervention [5]. However, a critical challenge persists in identifying the optimal delivery route to maximize therapeutic efficacy within the central nervous system (CNS).

Recent preclinical and clinical studies have demonstrated that MSCs can exert beneficial effects in neurodegenerative diseases, including AD [6, 7]. These benefits are believed to arise from a multifaceted mechanism involving the modulation of neuroinflammation, promotion of neuronal survival, and facilitation of Aβ clearance—partly through the activation of microglia [8, 9]. Notably, a phase 2a clinical trial of laromestrocel, an allogeneic bone marrow‐derived MSC product, demonstrated both safety and preliminary efficacy in slowing cognitive decline and brain atrophy in patients with mild AD, reinforcing the translational potential of MSC‐based interventions [8]. Such findings suggest that MSCs may provide therapeutic benefit through mechanisms that extend beyond direct targeting of amyloid and tau pathologies.

A critical determinant of therapeutic efficacy in MSC‐based CNS therapies is the route of administration, particularly in light of the restrictive nature of the blood–brain barrier (BBB) [9]. While intravenous (IV) administration is widely utilized due to its simplicity and minimally invasive nature, the BBB significantly restricts the transmigration of systemically administered cells into the brain parenchyma [10], potentially limiting therapeutic outcomes in neurodegenerative settings. Conversely, intrathecal (IT) administration, which involves direct delivery of cells into the cerebrospinal fluid, bypasses the BBB and facilitates more robust interactions between transplanted cells and CNS targets. Despite theoretical advantages, prior studies have largely been restricted to single‐route evaluations, and direct head‐to‐head comparisons of intravenous versus intrathecal efficacy under identical conditions remain scarce.

To address the knowledge gap concerning how distinct delivery routes (IT vs. IV) impact microglial modulation and downstream Aβ pathology, we conducted a direct comparative study in the well‐characterized 5xFAD transgenic mouse model. We comprehensively assessed outcomes across behavioral, histopathological, and molecular domains, including spatial learning and memory, amyloid plaque burden, microglial activation, and the structural and functional integrity of the microglial barrier. In addition, we examined the expression of key signaling molecules—triggering receptor expressed on myeloid cells 2 (TREM2), DNAX‐activating protein‐12 (DAP12, also known as TYROBP for tyrosine kinase binding protein), and spleen tyrosine kinase (Syk)—to investigate potential mechanisms underlying route‐dependent differences in efficacy. By integrating these multidimensional analyses, this study aims to inform optimal delivery strategies to maximize the therapeutic potential of MSC‐based interventions for AD.

## 2 Methods

### 2.1 Ethics Statement

All procedures involving human‐derived materials were conducted in accordance with the Declaration of Helsinki and were reviewed and approved by the Ethics Committee of Taihe Hospital, Hubei University of Medicine (Approval No. 2020XM004; approved on August 17, 2020). Human umbilical cords were obtained from healthy full‐term donors after written informed consent had been obtained.

All animal experiments were conducted in accordance with the Guidelines for the Care and Use of Laboratory Animals and were approved by the Animal Ethics Committee of Hubei University of Medicine (Approval No. 2018‐141; approved on December 30, 2018). The animal experiments and reporting were conducted in accordance with the ARRIVE 2.0 guidelines.

### 2.2 Animals

Transgenic 5xFAD mice and wild‐type (WT) controls were procured from The Jackson Laboratory. 5xFAD mice were bred with WT counterparts, and genotyping was performed to confirm offspring genotypes. Animals were maintained under specific pathogen‐free (SPF) conditions at 22°C ± 2°C and 50%–60% relative humidity, with a 12‐h light/dark cycle and ad libitum access to sterilized food and water.

### 2.3 Experimental Design and Grouping

Three‐month‐old 5xFAD and WT mice (20–22 g) were randomly allocated into six groups (*n* = 12 per group; six males and six females): WT control (WT), untreated 5xFAD control (5xFAD), 5xFAD with intravenous vehicle (5xFAD‐IV‐Vehicle), 5xFAD with intravenous HUCMSCs (5xFAD‐IV‐HUCMSCs), 5xFAD with intrathecal vehicle (5xFAD‐IT‐Vehicle), and 5xFAD with intrathecal HUCMSCs (5xFAD‐IT‐HUCMSCs). Randomization was conducted using the RAND() function in Microsoft Excel.

### 2.4 Experimental Procedures

The study comprised a two‐phase protocol: a treatment phase followed by an observation phase (Figure 1A). During the two‐month treatment phase, mice received weekly intravenous or intrathecal injections, totaling eight administrations. This was followed by a three‐month observation period. Behavioral assessments, including the Morris water maze, and histological analyses were conducted at the conclusion of the observation period by investigators blinded to group allocations.

**FIGURE 1:**
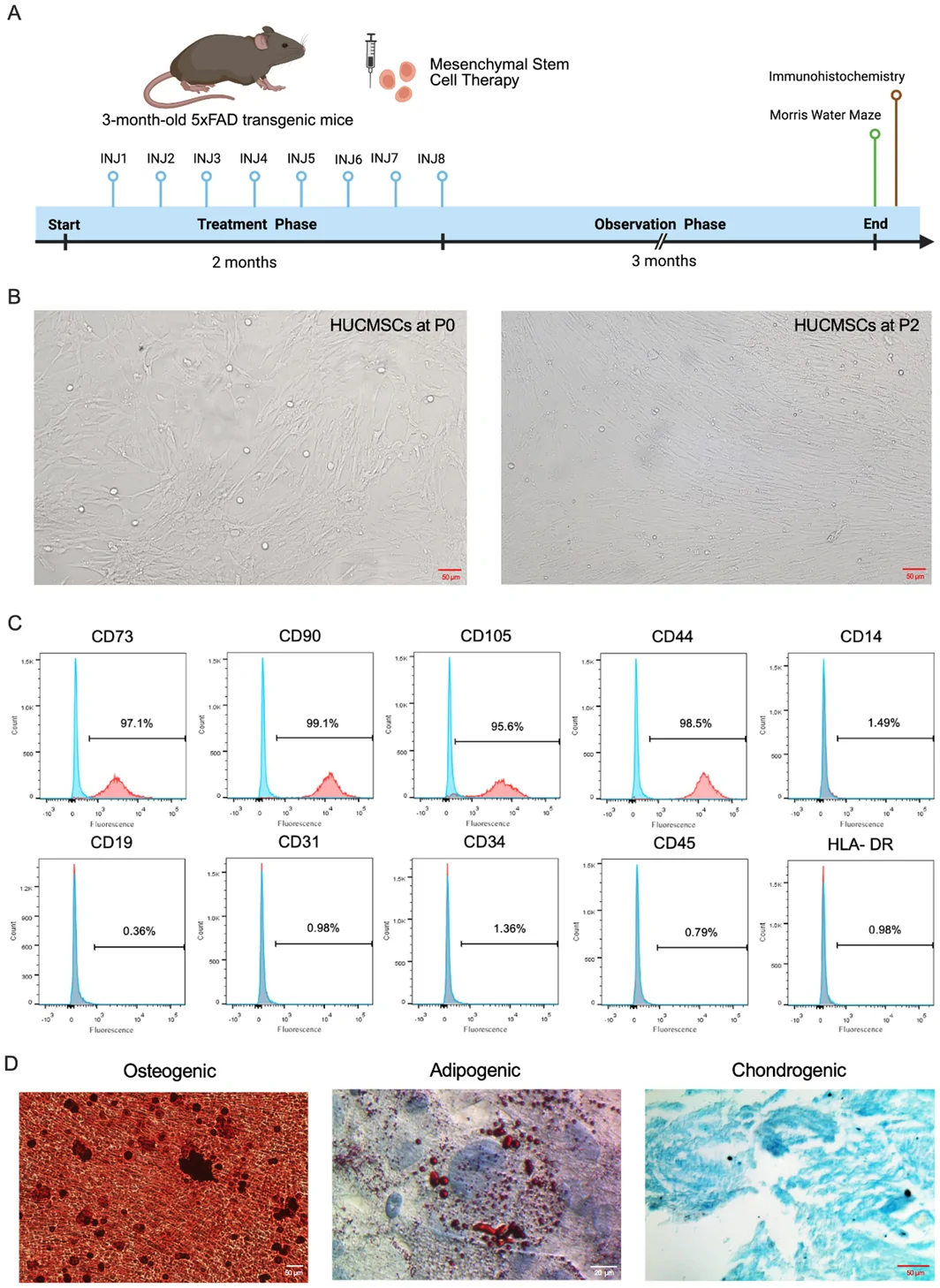
Characterization of HUCMSCs. (A) Schematic illustration of experimental design. (B) Light Microscopy Image of HUCMSCs. Cells were observed migrating from the edge of Wharton's jelly, exhibiting a characteristic spindle‐shaped morphology and radial arrangement. (C) Flow cytometric analysis of HUCMSC surface markers. (D) Multilineage differentiation capacity of HUCMSCs. Cells successfully differentiated into osteoblasts (Alizarin Red staining, left panel, 100×), adipocytes (Oil Red O staining, middle panel, 400×), and chondrocytes (Alcian Blue staining, right panel, 200×).

Recognizing the immunological challenges associated with xenogeneic transplantation—specifically, the rapid clearance of HUCMSCs by the murine immune system—we deliberately opted not to use immunosuppressive agents due to their well‐documented clinical limitations and potential adverse effects. Instead, we employed a compensatory strategy involving an increased frequency and duration of HUCMSC administration (eight injections over two months) to mitigate immune‐mediated clearance and sustain therapeutic cell presence.

### 2.5 Isolation and Characterization of HUCMSCs

HUCMSCs were isolated via the tissue explant adherence method [11]. Umbilical cords were thoroughly washed, minced, and cultured in α‐ Minimum Essential Medium (MEM, HyClone, USA) supplemented with 10% fetal bovine serum (FBS, Gibco, USA), 2.5 ng/mL basic fibroblast growth factor (bFGF, Gibco, USA), and 20 ng/mL epidermal growth factor (EGF, Gibco, USA). Cultures were maintained at 37°C in a 5% CO_2_ atmosphere, and cells were harvested upon reaching confluence. Flow cytometry confirmed the expression of MSC‐specific markers (CD73, CD90, CD105, CD44; 1:1000, BioLegend, USA) and the absence of hematopoietic markers (CD14, CD19, CD31, CD34, CD45, HLA‐DR; 1:1000, BioLegend, USA). Multipotent differentiation potential was verified by inducing osteogenic, adipogenic, and chondrogenic lineages under defined culture conditions.

### 2.6 HUCMSC Transplantation

Cell viability was assessed using the xCELLigence Real‐Time Cell Analysis (RTCA) system (Agilent Technologies, USA) prior to transplantation [12]. HUCMSCs were administered via intravenous (IV; 5 × 10^7^ cells/kg in 100 μL normal saline, tail vein) or intrathecal (IT; 1 × 10^7^ cells/kg in 10 μL normal saline) routes. Injections were performed once weekly for eight consecutive weeks.

For IT injections, a modified 29G needle with a 90° bend (0.5 cm from the tip) was employed under sterile conditions [13]. Mice were positioned prone with lumbar flexion to expose the L5‐L6 intervertebral space. Following povidone‐iodine disinfection, the needle was inserted at a 100° angle to the spinal axis. Subarachnoid entry was confirmed by observing a tail flick reflex, after which the needle was rotated parallel to the spine and advanced 5 mm.

### 2.7 Morris Water Maze Behavioral Assessment

Spatial learning and memory were evaluated using the Morris water maze. The apparatus consisted of a circular pool (120 cm diameter, 50 cm depth) filled with opaque water maintained at 22°C–25°C. A hidden platform (10 cm diameter) was submerged 1 cm below the water surface. Visual cues and standardized lighting (430 lx) provided spatial orientation. Mice underwent a five‐day training period (three trials per day, randomized entry points, 60 s cutoff per trial). Twenty‐four hours after the final training session, a probe trial was conducted (60 s free swim with the platform removed) to assess memory retention, with metrics including the time spent in the target quadrant and the total swimming distance within that quadrant. All behavioral testing and analyses were performed by blinded investigators.

### 2.8 Immunofluorescence

Mice were deeply anesthetized with intraperitoneal sodium pentobarbital (50 mg/kg) and perfused transcardially with phosphate‐buffered saline (PBS), followed by 4% paraformaldehyde (PFA) for fixation. Brains were post‐fixed overnight at 4°C and sectioned coronally (40 μm) using a Leica VT1000S vibratome. Sections were incubated with primary antibodies in PBS containing 0.2% Triton X‐100 and 5% goat serum for 72 h at 4°C, followed by secondary antibody incubation for 6 h. This extended incubation protocol was employed to enhance antibody penetration and fluorescence signal intensity in thick free‐floating sections [14]. Amyloid plaques were visualized using Thioflavin S staining (0.0003% w/v, 15 min incubation, room temperature). Primary antibodies included anti‐Ionized calcium‐binding adapter molecule 1 (anti‐Iba‐1, 1:500, Wako, Japan), Anti‐Lysosomal Associated Membrane Protein 1 (anti‐LAMP‐1, 1:500, DSHB, USA), anti‐TREM2 (1:500, R&D Systems, USA), anti‐DAP12 (1:500, LSBio, USA), and anti‐ Syk (1:500, Sigma–Aldrich, USA).

### 2.9 Confocal Microscopy and Image Analysis

Confocal images were acquired using an Olympus FV3000 laser‐scanning microscope equipped with a motorized stage. To enable precise anatomical localization of the hippocampus and cortex for subsequent high‐magnification imaging, tile‐scan images of the hemibrain sections were first acquired using a 10× objective (NA = 0.45). All acquisition parameters for Thioflavin‐S (ThS) and Iba1 immunofluorescence—including laser power, detector gain, offset, pinhole size, pixel dwell time, and scanning speed—were kept identical across experimental groups within each staining batch to ensure strict comparability. For high‐resolution qualitative and quantitative analyses, z‐stack images were acquired using a 60× oil‐immersion objective (NA = 1.42) at a 1‐μm step size and 1024 × 1024 pixel resolution. Maximum‐intensity projections of 10 central optical slices were generated for downstream measurements. Within each high‐power field (60×), both the total number and mean fluorescence intensity of amyloid plaques and microglia were quantified.

Three‐dimensional image reconstruction and automated quantitative analyses were performed using Matrix Laboratory (MATLAB). Amyloid plaque boundaries were delineated by applying an intensity threshold set at two standard deviations above the mean background fluorescence. Plaque size measurements were derived from *z*‐axis maximum‐intensity projections of five optical slices centered on the equatorial plane of each plaque. Microglial barrier‐related parameters were evaluated as previously described [15]. Using an automated MATLAB script, the number of microglia located within 15‐ and 30‐μm radii of each plaque was determined. Microglial coverage was calculated as the proportion of the plaque perimeter overlapped by Iba1‐positive microglial processes.

### 2.10 Bioinformatics Analysis

Between 2000 and 2021, four Gene Expression Omnibus (GEO) datasets (GSE115437, GSE161848, GSE97113, and GSE134151) were retrieved from the National Center for Biotechnology Information (NCBI) GEO database using the keywords “5xFAD,” “Wild type,” and “Hippocampus.” Among these, only GSE161848 was found to contain complete hippocampal transcriptomic data and Supporting Information suitable for subsequent analysis. Data preprocessing, including quality control and normalization, was conducted in R. Differentially expressed genes (DEGs) were identified using the limma package, applying thresholds of *p* < 0.05 and |log_2_FC| > 1. Functional enrichment analyses of DEGs were performed using the Enrichr platform, focusing on Gene Ontology (GO) terms and WikiPathways [16]. Data visualization was accomplished using R packages ggplot2 and pheatmap to generate volcano plots and heatmaps.

### 2.11 Statistical Analysis

All data are presented as mean ± SEM. Statistical analyses were conducted using SPSS (IBM) and GraphPad Prism (GraphPad Software). For the Morris water maze training phase, escape latency and swimming distance across training days were analyzed using two‐way repeated measures Analysis of variance (ANOVA), with group and training day as factors, followed by Fisher's Least Significant Difference (LSD) post hoc test when appropriate. Probe trial performance, swimming speed, and all other behavioral, histological, and molecular data were analyzed using one‐way ANOVA with Fisher's LSD post hoc test or two‐tailed unpaired *t*‐tests, as appropriate. Statistical significance was defined as *p* ≤ 0.05.

## 3 Results

### 3.1 Characterization and Viability Assessment of HUCMSCs

HUCMSCs were identified based on their morphology, surface marker expression, and multipotent differentiation capacity. Morphologically, the cells exhibited adherence, spreading, and a radial growth pattern (Figure 1B). Flow cytometric analysis confirmed that the cultured cells met the International Society for Cellular Therapy (ISCT) criteria for MSCs, demonstrating high expression (> 95%) of MSC markers CD73, CD90, CD105, and CD44, while exhibiting negligible (< 2%) expression of negative markers including CD14, CD34, CD45, and Human Leukocyte Antigen‐DR isotype (HLA‐DR) (Figure 1C). Furthermore, the cells successfully differentiated into osteogenic, adipogenic, and chondrogenic lineages under specific induction conditions (Figure 1D), confirming their multipotency.

To ensure the viability and functional integrity of HUCMSCs prior to transplantation, real‐time monitoring was conducted using the xCELLigence RTCA system. The cells exhibited a typical three‐phase growth profile: an initial adhesion phase with a gradual increase in cell index (CI), followed by exponential proliferation, and a subsequent plateau indicative of confluence. These results confirmed robust cell viability before transplantation (Figure S1).

### 3.2 HUMSCs Transplantation Improves Cognitive Deficits in 5xFAD Mice

To assess the therapeutic efficacy of different transplantation routes, spatial learning and memory were evaluated using the Morris water maze. WT controls significantly outperformed 5xFAD controls on Days 4 and 5 of training (*p* < 0.001), validating cognitive impairment in the 5xFAD model (Figure 2). Among 5xFAD groups, the 5xFAD‐IT‐HUCMSC group demonstrated significantly improved performance relative to vehicle controls (*p* < 0.05). 5xFAD‐IV‐HUCMSC group also improved performance, though not significantly.

**FIGURE 2:**
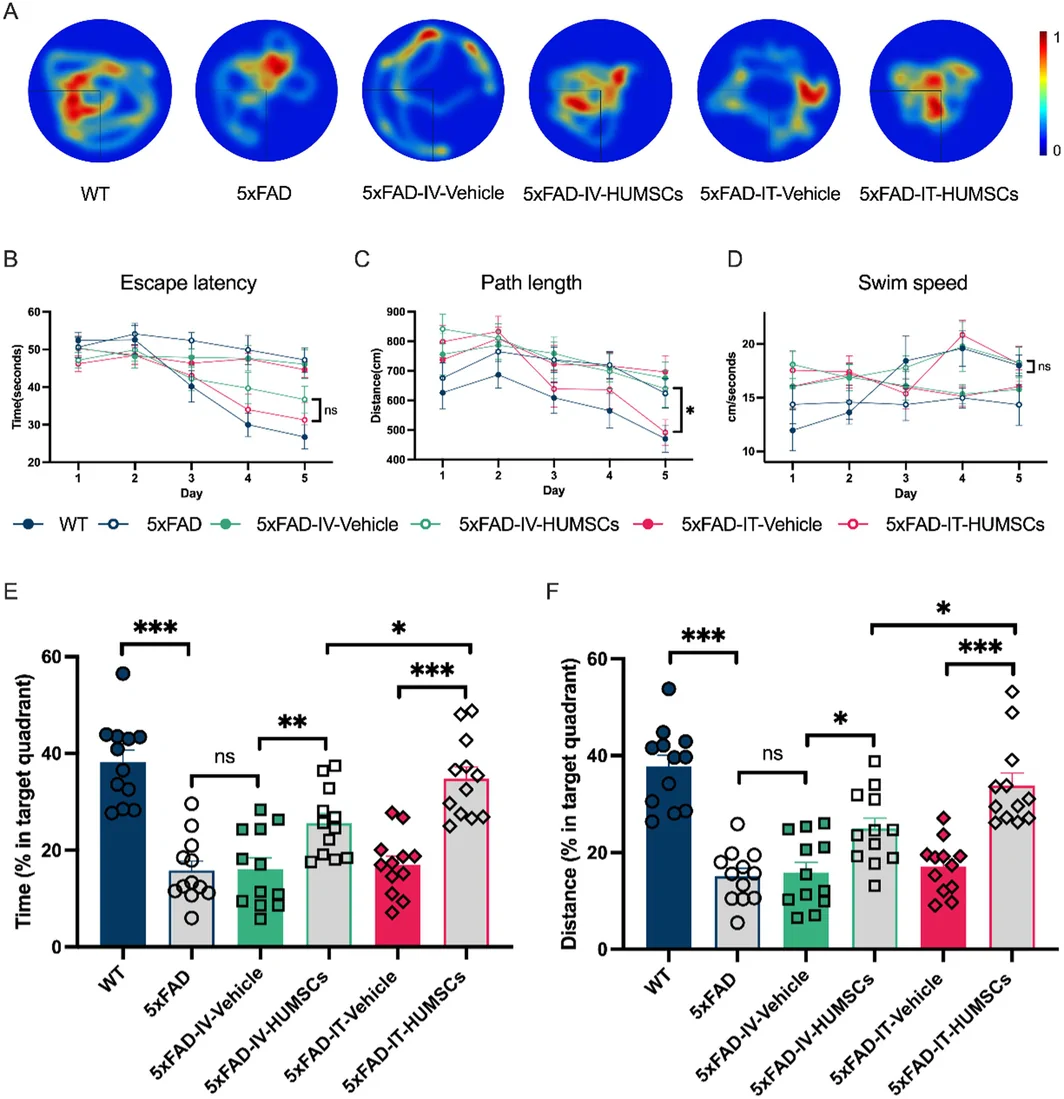
Effects of HUCMSC transplantation via intravenous (IV) and intrathecal (IT) routes on spatial learning and memory in 5xFAD mice. (A) Representative heat maps showing swimming trajectories during the probe trial of the Morris water maze test. (B) Escape latency during the 5‐day training phase. (C) Total swimming distance during the training phase. (D) Swimming speed during the training phase. Statistical comparisons between the IV and IT HUCMSC transplantation groups on day 5 are indicated in panels B–D. (E) Time spent in the target quadrant during the probe trial. (F) Distance traveled in the target quadrant during the probe trial. Data are presented as mean ± SEM (*n* = 12 per group). *p < 0.05, **p < 0.01 and ***p < 0.001.

On Day 5, total swimming distance was significantly reduced in the 5xFAD‐IT‐HUCMSC group compared to both the IT‐vehicle and 5xFAD‐IV‐HUCMSC groups (*p* < 0.05), indicating enhanced spatial learning with intrathecal delivery (Figure 2A,B). In probe trials, both HUCMSC‐treated groups showed improved memory retention compared to controls (*p* < 0.05), with intrathecal treatment being more effective (*p* < 0.05) (Figure 2D,E). No significant differences in swimming speed were observed among groups (Figure 2C). These results suggest that HUCMSC transplantation ameliorates cognitive deficits in 5xFAD mice, with intrathecal administration offering superior benefits.

To assess potential sex‐related differences in cognitive performance, sex‐stratified subgroup analyses were performed for Morris water maze–based behavioral outcomes (Figure S2). Overall, the trend of treatment effects on cognitive deficits was consistent between male and female mice. However, female 5xFAD mice generally exhibited more pronounced cognitive impairment than their male counterparts. Due to the relatively limited sample size within each sex‐specific subgroup, the statistical significance of certain comparisons was reduced, although the overall behavioral trends remained comparable between sexes.

### 3.3 HUCMSC Transplantation Reduces Amyloid Plaque Burden in 5xFAD Mice

We next evaluated amyloid plaque deposition in the hippocampus and cortex. No plaques were detected in WT mice (Figure 3). In 5xFAD mice, plaque number and size did not differ significantly among vehicle‐treated groups. Both intravenous and intrathecal HUCMSC treatments significantly reduced plaque number and size compared to vehicle controls (*p* < 0.05). Notably, intrathecal administration exhibited superior efficacy over intravenous delivery (*p* < 0.05).

**FIGURE 3:**
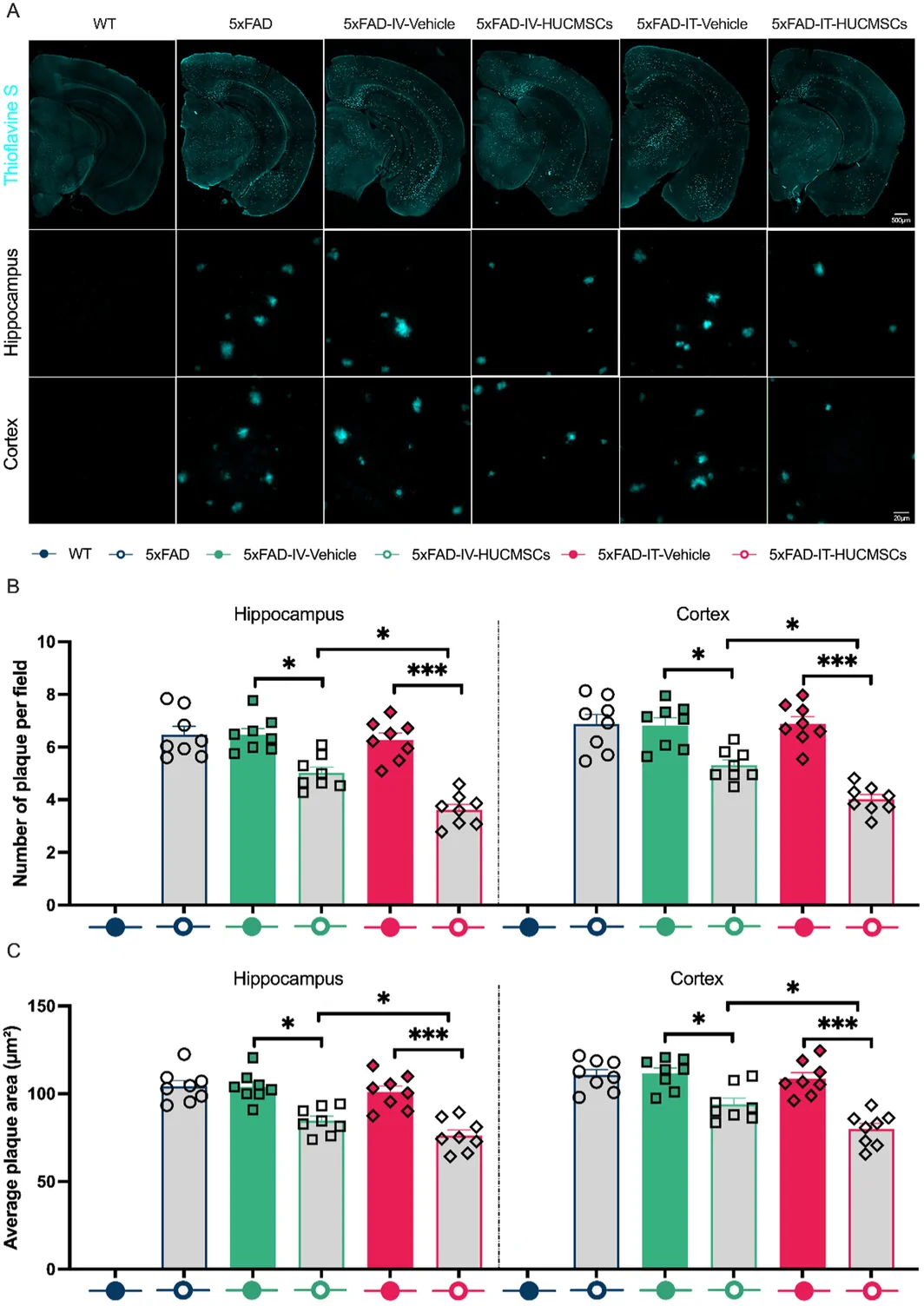
Effects of HUCMSC transplantation via intravenous (IV) and intrathecal (IT) routes on amyloid plaque burden in the hippocampus and cortex of 5xFAD mice. (A) Representative confocal images showing Thioflavin‐S (ThS)‐labeled amyloid plaques in the hippocampus and cortex across all experimental groups. Low‐magnification hemibrain images were acquired at 100×; scale bar = 500 μm. High‐magnification images were acquired at 600×; scale bar = 20 μm. (B) Quantification of the number of ThS‐positive amyloid plaques per high‐power microscopic field. (C) Quantitative analysis of average amyloid plaque sizes in the indicated brain regions. Data are presented as mean ± SEM (*n* = 8 mice per group; 40 fields analyzed per mouse). *p < 0.05 and ***p < 0.001.

To further evaluate potential sex‐related differences in amyloid pathology, sex‐specific subgroup analyses were performed (Figure S3). Overall, the trend of treatment‐related effects was comparable between male and female mice. However, female Alzheimer's disease mice tended to exhibit a greater amyloid‐β plaque burden than their male counterparts.

### 3.4 Enhanced Microglial Density and Fluorescence Intensity Following HUCMSC Treatment

Quantitative analysis revealed that microglial cell counts and fluorescence intensities were significantly lower in WT mice than in 5xFAD control and HUCMSC‐treated groups within both the hippocampus and cortex (Figure 4). Among 5xFAD mice, no significant difference was observed between control and vehicle groups (*p* > 0.05). HUCMSC treatment significantly increased microglial cell density and fluorescence intensity (*p* < 0.05, *p* < 0.001), with intrathecal administration showing superior efficacy compared to intravenous delivery (*p* < 0.05). These results indicate that HUCMSC therapy enhances microglial activation, particularly via the intrathecal route.

**FIGURE 4:**
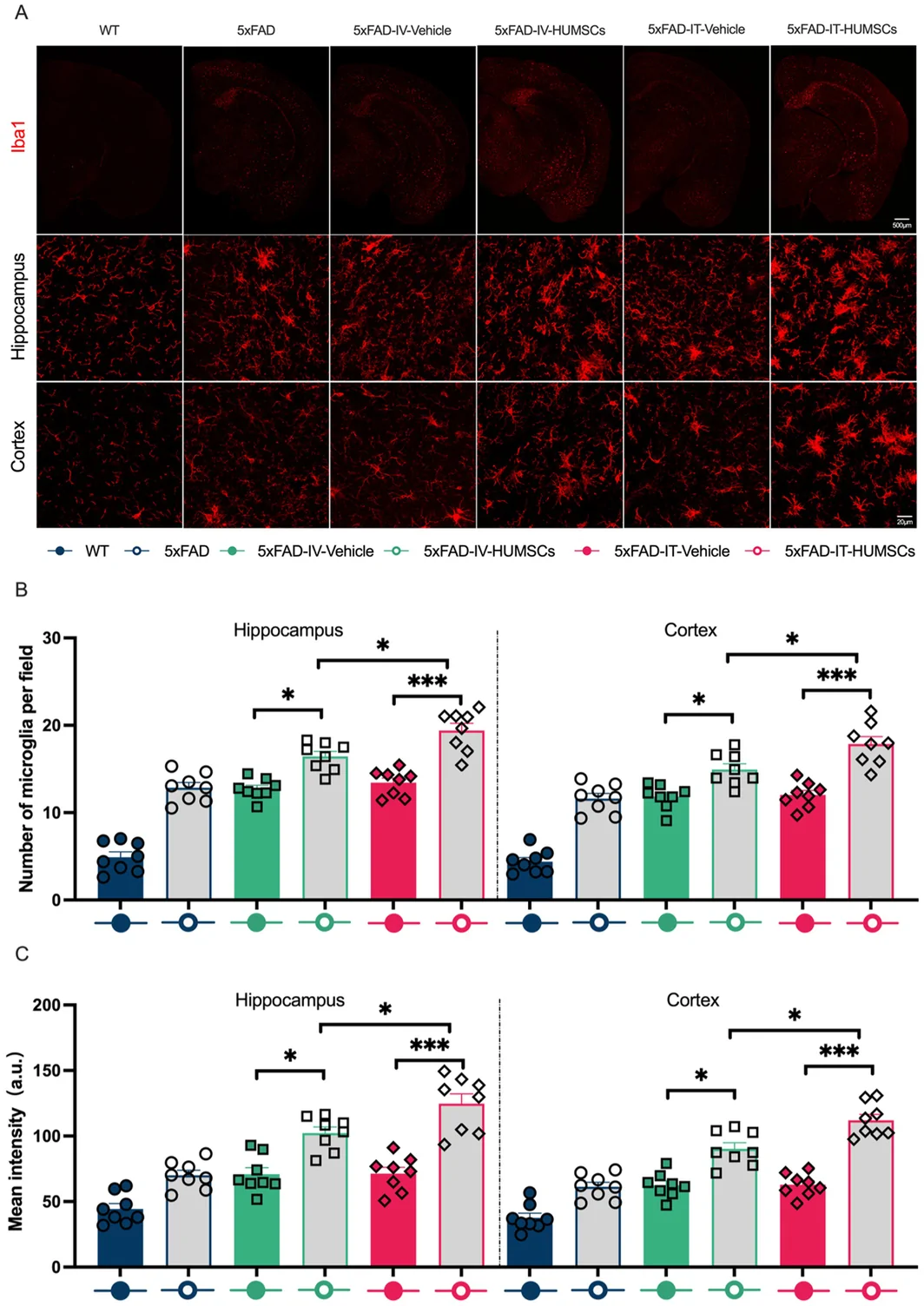
Effects of HUCMSC transplantation via intravenous (IV) and intrathecal (IT) routes on microglial cell number and Iba1 fluorescence intensity in the hippocampus and cortex of 5xFAD mice. (A) Representative confocal images showing Iba1‐labeled microglia in the hippocampus and cortex across all experimental groups. Low‐magnification hemibrain images were acquired at 100×; scale bar = 500 μm. High‐magnification images were acquired at 600×; scale bar = 20 μm. (B) Quantification of the number of Iba1‐positive microglia per field. (C) Quantification of the mean fluorescence intensity of Iba1‐positive microglia. Data are expressed as mean ± SEM (*n* = 8 mice per group; 40 fields analyzed per mouse). *p < 0.05 and ***p < 0.001.

### 3.5 HUCMSC Treatment Enhances Plaque Compaction and Microglial Coverage

To investigate the effects of HUCMSCs on microglial barrier formation and plaque compaction, we quantified microglial coverage and plaque density. Microglia form a protective barrier around plaques, modulating plaque morphology and reducing neurotoxicity [15, 17]. Vehicle‐treated groups showed no significant differences in microglial coverage or plaque density. In contrast, HUCMSC treatment significantly increased both microglial coverage and plaque density in hippocampal and cortical regions (*p* < 0.05, *p* < 0.001), with intrathecal administration demonstrating superior efficacy over intravenous delivery (*p* < 0.05) (Figure 5).

**FIGURE 5:**
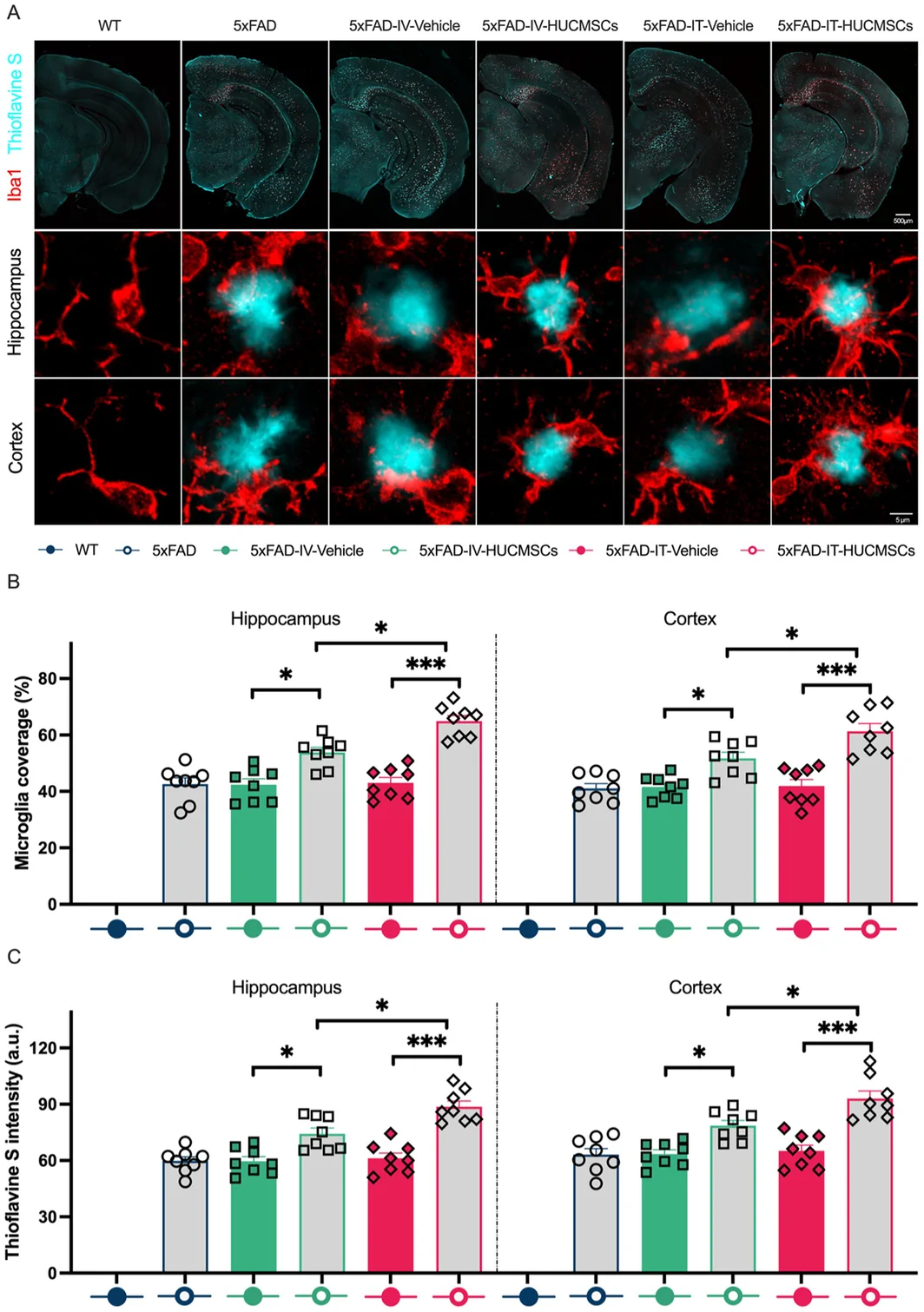
Effects of HUCMSC transplantation via intravenous (IV) and intrathecal (IT) routes on microglial barrier coverage and plaque density in 5xFAD mice. (A) Representative confocal images showing microglial coverage surrounding amyloid plaques in the hippocampus and cortex across all experimental groups. Low‐magnification hemibrain images were acquired at 100×; scale bar = 500 μm. High‐magnification images were acquired at 600×; scale bar = 5 μm. (B) Quantification of microglial coverage around amyloid plaques. (C) Quantification of plaque density. Data are presented as mean ± SEM (*n* = 8 mice per group). Total number of plaques analyzed: 1254 (5xFAD control), 1227 (5xFAD‐IV‐Vehicle), 1787 (5xFAD‐IV‐HUCMSCs), 1359 (5xFAD‐IT‐Vehicle), and 2453 (5xFAD‐IT‐HUCMSCs). *p < 0.05 and ***p < 0.001.

### 3.6 HUCMSC Transplantation Attenuates Axonal Dystrophy in Microglial Barrier Regions

Axonal dystrophy surrounding amyloid plaques was assessed using Lamp1 immunostaining, a marker of lysosome‐enriched dystrophic neurites [15]. Both HUCMSC‐treated groups exhibited significantly reduced Lamp1‐positive axonal dystrophy in hippocampal and cortical regions compared to controls (*p* < 0.05). Intrathecal treatment resulted in a smaller Lamp1‐positive area than intravenous treatment (*p* < 0.05), although differences in Lamp1 fluorescence intensity did not reach statistical significance (Figure 6). These results indicate that HUCMSC transplantation mitigates axonal dystrophy, with intrathecal administration providing superior structural neuroprotection.

**FIGURE 6:**
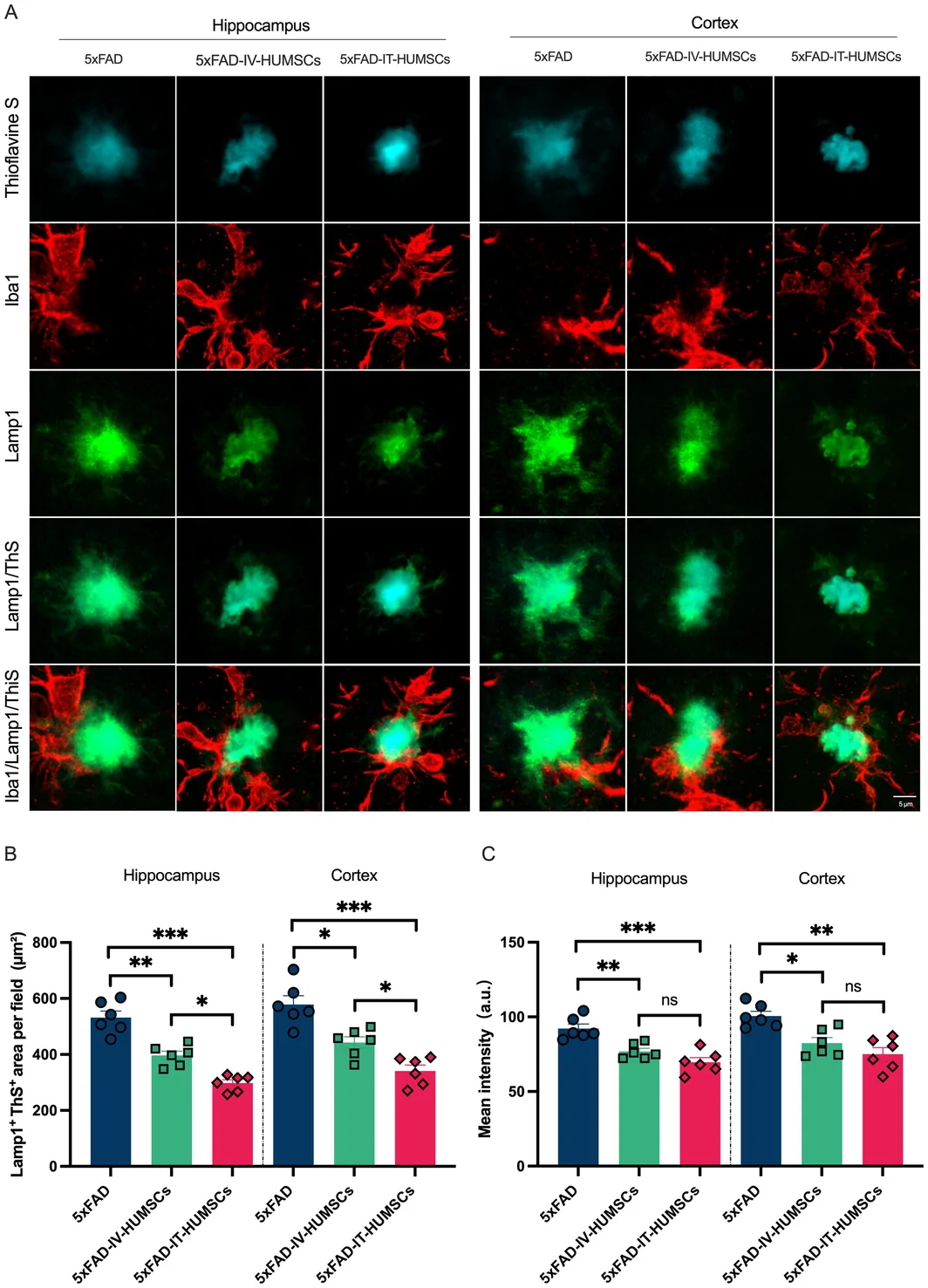
Effects of HUCMSC transplantation via intravenous (IV) and intrathecal (IT) routes on plaque‐associated dystrophic neurite pathology in 5xFAD mice. (A) Representative confocal images showing LAMP1‐positive dystrophic neurite pathology surrounding amyloid plaques across experimental groups. High‐magnification images were acquired at 600×; scale bar = 5 μm. (B) Quantification of LAMP1‐positive areas in the hippocampus and cortex. (C) Quantification of mean LAMP1 fluorescence intensity in the hippocampus and cortex. Data are expressed as mean ± SEM (*n* = 6 mice per group; 40 fields analyzed per mouse). *p < 0.05, **p < 0.01 and ***p < 0.001.

### 3.7 Bioinformatics Analysis of Microglial Activation Pathways

To elucidate the molecular mechanisms underlying HUCMSC‐mediated microglial modulation, we performed bioinformatics analyses using publicly available transcriptomic datasets. A total of 129 genes were significantly upregulated in 5xFAD mice (adjusted *p* < 0.05), with Trem2 and TYROBP (DAP12) ranking among the top 15 (Figure S4). Enrichment analyses revealed that these genes were predominantly associated with microglial activation, phagocytosis, and neuroinflammatory pathways. WikiPathways analysis further highlighted enrichment in pathways related to pathogen phagocytosis and complement activation. Based on these results, TREM2 and DAP12 were selected for further validation.

### 3.8 Upregulation of Trem2 Expression Following HUCMSC Treatment

To evaluate the involvement of the TREM2/DAP12 pathway in microglial activation and barrier formation, we performed immunofluorescence staining using anti‐TREM2 antibodies alongside Thioflavin S. TREM2 expression was significantly upregulated in both HUCMSC‐treated groups compared to controls (*p* < 0.05), with intrathecal administration demonstrating superior efficacy (*p* < 0.05). TREM2 fluorescence intensity was also elevated in both treatment groups, with no significant difference between administration routes (Figure S5).

To determine the cellular specificity of TREM2 upregulation, quantitative co‐localization analysis was performed using double immunofluorescence staining for TREM2 and the microglial marker Iba1 (Figure S6). The majority of TREM2‐positive signals co‐localized with Iba1‐positive microglia, indicating that the increased TREM2 expression observed following HUCMSC treatment predominantly occurred in microglial cells.

### 3.9 Increased DAP12 Expression Following HUCMSC Treatment

Given that DAP12 functions downstream of TREM2 to mediate microglial signaling, we next examined its expression. Both intravenous and intrathecal HUCMSC treatments significantly increased DAP12‐positive areas and fluorescence intensity compared to vehicle controls (*p* < 0.05). Intrathecal administration yielded a greater increase in DAP12‐positive area than intravenous delivery (*p* < 0.05), although differences in fluorescence intensity between the two treatment groups were not statistically significant (Figure S7). These findings might suggest that HUCMSC transplantation, particularly via the intrathecal route, enhances microglial activation through the TREM2/DAP12 axis.

To determine the cellular localization of the increased DAP12 signal, we performed quantitative co‐localization analysis using dual immunofluorescence staining for DAP12 and Iba1. The majority of DAP12‐positive signals co‐localized with Iba1‐positive microglia (Figure S8), indicating that the HUCMSC treatment‐associated increase in DAP12 expression occurred predominantly in microglia.

### 3.10 HUCMSC Treatment Enhanced Syk Activation in 5xFAD Mice

To further validate downstream signaling, we assessed activation of Syk, a key effector of the TREM2/DAP12 pathway. Immunofluorescence analysis showed significantly increased Syk‐positive areas in both intravenous (*p* < 0.01) and intrathecal (*p* < 0.001) HUCMSC‐treated groups compared to controls (Figure S9). These results might suggest that HUCMSC transplantation activates Syk‐mediated signaling, with intrathecal administration inducing more robust effects.

To determine the cellular localization of the increased Syk signal, we performed dual immunofluorescence staining for Syk and Iba1. Quantitative co‐localization analysis showed that the majority of Syk‐positive signals co‐localized with Iba1‐positive microglia (Figure S10), indicating that the HUCMSC treatment‐associated increase in Syk expression occurred predominantly in microglia.

## 4 Discussion

In this study, we evaluated the therapeutic efficacy of HUCMSCs in a transgenic AD mouse model (5xFAD), with a particular focus on the impact of delivery route. Our results yielded several important findings: (1) HUCMSC transplantation significantly improved spatial learning and memory in 5xFAD mice, with intrathecal delivery showing superior efficacy over intravenous administration; (2) HUCMSC treatment reduced amyloid plaque burden and axonal dystrophy, while enhancing plaque compaction and microglial barrier formation; and (3) these effects were accompanied by increased microglial activation and upregulation of TREM2, DAP12, and Syk, suggesting activation of a protective microglial signaling cascade.

The observed improvements in cognitive performance are consistent with prior studies demonstrating the neurocognitive benefits of MSC‐based therapies in AD models [8, 18]. These findings align with a recent phase 2a clinical trial of laromestrocel, an intravenously administered allogeneic bone marrow‐derived MSC product, which showed preliminary efficacy in slowing cognitive decline and brain atrophy in patients with mild AD [8]. Our study reinforces these clinical insights and extends them by directly comparing delivery routes in a preclinical model, thereby addressing a key translational knowledge gap.

While intravenous infusion remains the most commonly employed route in clinical settings due to its minimally invasive nature, its therapeutic efficacy in CNS diseases is limited by the BBB, which restricts peripheral cell migration into brain parenchyma [9]. In contrast, intrathecal administration bypasses the BBB and facilitates direct access of transplanted cells to the cerebrospinal fluid, enabling closer proximity to pathological substrates. This may account for the significantly greater cognitive improvements, reduced swimming distances, and enhanced memory retention observed in the intrathecal HUCMSC group compared to the intravenous group.

In addition to behavioral benefits, our findings show that HUCMSC treatment reduced amyloid plaque burden and dystrophic neurites, as evidenced by lower plaque counts, decreased plaque size, and diminished Lamp1‐positive axonal dystrophy in both the hippocampus and cortex. Notably, intrathecal delivery outperformed intravenous administration in reducing dystrophic pathology, suggesting more effective CNS engagement. These results reinforce previous findings that MSCs can promote Aβ clearance and protect against synaptic and axonal damage [6, 7, 8].

We also demonstrate that HUCMSCs enhanced microglial activation and functional transformation, as shown by increased microglial density, higher fluorescence intensity, and greater microglial coverage of plaques. The formation of the microglial barrier—a protective structure that physically isolates amyloid plaques—is essential for mitigating Aβ‐induced neurotoxicity [15, 17, 19]. Our data reveal that HUCMSCs significantly enhanced both microglial coverage and plaque compaction, especially via intrathecal delivery, indicating an augmented protective microglial response.

At the molecular level, we observed that HUCMSC treatment upregulated TREM2, DAP12, and Syk expression—key components of the microglial activation cascade. The TREM2/DAP12 signaling axis plays a pivotal role in microglial barrier formation, facilitating plaque compaction, limiting amyloid fibril spread, and insulating nearby neurons from neurotoxic insults [15, 17, 19, 20]. Our findings not only validate the critical role of this pathway but also provide novel evidence that intrathecal administration amplifies TREM2/DAP12/Syk signaling more effectively than intravenous delivery, thereby enhancing microglial‐mediated amyloid clearance. This highlights the importance of delivery route optimization in maximizing the therapeutic efficacy of MSC‐based interventions for AD.

A key strength of this study is its direct head‐to‐head comparison of intrathecal versus intravenous HUCMSC administration, which demonstrates that intrathecal delivery yields superior therapeutic outcomes across behavioral, histopathological, and molecular domains by more effectively enhancing local microglial activation and amyloid‐β clearance, despite a lower total cell dose—thereby providing critical preclinical and translational insight for optimizing administration strategies in stem cell–based therapies for neurodegenerative disorders.

Despite these promising results, several limitations must be acknowledged. First, this study was conducted using a single transgenic AD model (5xFAD), which, although well‐characterized and widely validated, does not fully recapitulate the complexity of sporadic AD in humans—most notably, it exhibits virtually no tau pathology, and tau‐related changes were therefore not assessed in this work. Validation in additional AD models, including tauopathy models and age‐related models, will be necessary. Second, xenogeneic HUCMSCs were transplanted without immunosuppression, a choice that may have limited cell survival. However, immunosuppressive regimens were intentionally avoided, as they introduce substantial confounding effects and diminish translational relevance. This approach was adopted to better approximate a clinically realistic therapeutic scenario. In addition, our observations reflect relatively short‐term outcomes, and the long‐term durability, biodistribution, and safety of repeated HUCMSC administration remain unknown. The absence of in vivo cell tracking represents a further limitation; nevertheless, we are currently preparing a dedicated follow‐up study focused on HUCMSC biodistribution and persistence, which will be submitted separately. Longitudinal investigations with extended follow‐up will be essential to determine the sustainability of therapeutic effects and to assess the risk of delayed adverse events. Finally, although intrathecal administration consistently demonstrated superior therapeutic efficacy compared with intravenous delivery, the mechanisms underlying this advantage were not directly interrogated in the present work. Whether intrathecal delivery enables more efficient penetration into the brain parenchyma, enhanced cell survival or bioactivity, or distinct differentiation or paracrine signaling profiles of HUCMSCs remains unclear and warrants further mechanistic investigation. Moreover, while no overt adverse effects were observed, we recognize that repeated intrathecal injections may pose a risk of local or meningeal inflammation, an issue that should be carefully evaluated in future preclinical and translational studies.

## 5 Conclusions

This study demonstrates that HUCMSC transplantation, particularly via intrathecal administration, significantly improves cognitive function, reduces amyloid pathology, and enhances microglial barrier integrity in the 5xFAD transgenic mouse model of AD. These findings highlight the therapeutic potential of HUCMSCs and underscore the critical importance of delivery route optimization in the development of effective stem cell‐based interventions for neurodegenerative disorders such as AD.

## Author Contributions

L.D., W.Y., and Y.C. participated in the culture of mesenchymal stem cells, animal experiments, and data analysis. L.D. drafted the manuscript. H.W. contributed to data visualization, bioinformatics analysis, and manuscript revision. S.L. and H.L. were involved in bioinformatics analysis and critical revision of the manuscript. T.S., X.H., Y.Y., and R.L. participated in portions of the animal experiments. X.L. performed tail vein injections. Z.A. and X.G. provided guidance during the animal experiments. Q.Q., J.C., and L.D. contributed to the study design. All authors participated in data interpretation and critical revision of the manuscript.

## Funding

This study was supported by the Health Commission of Hubei Province Scientific Research Project (no. ZY2019M047), the Science and Technology Research and Development Project Plan of Shiyan City (no. 2020K52), the Stem Cell Research Fund of Taihe Hospital (no. GXB2023004), the Health Commission of Hubei Province Traditional Chinese Medicine Research Project for Youths (no. ZY2023Q049), Natural Science Foundation of Hubei Province (no. 2023AFB336).

## Consent

All authors have given consent for this publication.

## Conflicts of Interest

The authors declare no conflicts of interest.

## Supporting information


**Figure S1:** Real‐time cell analysis (RTCA) growth curve of HUCMSCs. Dynamic changes in the Cell Index (CI) of HUCMSCs were monitored over a 96‐h culture period using the RTCA system. The curve shows the dynamic growth profile of HUCMSCs over time. Data are presented as mean ± SEM from five independent experiments (*n* = 5).
**Figure S2:** Sex‐stratified analysis of Morris water maze performance in 5xFAD mice. (A–C) Morris water maze performance in male mice. (D–F) Morris water maze performance in female mice. (A and D) Escape latency during the 5‐day training phase. (B and E) Total swimming distance during the training phase. (C and F) Swimming speed during the training phase. (G) Time spent in the target quadrant during the probe trial. (H) Distance traveled in the target quadrant during the probe trial. Data are expressed as mean ± SEM (*n* = 6 mice per group). *p < 0.05, **p < 0.01 and ***p < 0.001.
**Figure S3:** Sex‐stratified analysis of amyloid‐β plaque burden in the hippocampus and cortex of 5xFAD mice. (A and C) Quantification of amyloid‐β plaque burden in the hippocampus. (B and D) Quantification of amyloid‐β plaque burden in the cortex. (A and B) Number of Thioflavin‐S (ThS)‐positive amyloid plaques per high‐power field. (C and D) Average size of ThS‐positive amyloid plaques in the indicated brain regions. Data are presented as mean ± SEM (*n* = 4 mice per group; 40 fields analyzed per mouse). *p < 0.05, **p < 0.01 and ***p < 0.001.
**Figure S4:** Bioinformatic analysis of differentially expressed genes (DEGs) and related pathways. (A) Volcano plot showing DEGs. The top 15 upregulated genes are labeled in red. Dashed lines indicate the significance thresholds: |log_2_FC| > 1 and adjusted *p* < 0.05. (B) Hierarchical clustering heatmap of the top 50 DEGs. Red indicates upregulated genes, and blue indicates downregulated genes. (C) Gene Ontology (GO) enrichment analysis of the top 10 biological processes associated with DEGs. (D) GO enrichment analysis showing representative biological processes related to microglial activation, phagocytosis, and neuroinflammatory signaling, together with associated DEGs. (E) WikiPathways enrichment analysis showing representative pathways associated with DEGs. (F) Identification of key genes, including TREM2 and TYROBP/DAP12, based on their enrichment across biological processes and pathways.
**Figure S5:** Effects of HUCMSC transplantation via intravenous (IV) and intrathecal (IT) routes on TREM2 expression in 5xFAD mice. (A) Representative confocal images showing TREM2 immunofluorescence in the hippocampus and cortex across experimental groups. High‐magnification images were acquired at 600×; scale bar = 5 μm. (B) Quantification of TREM2‐positive area around amyloid plaques. (C) Quantification of mean TREM2 fluorescence intensity. Data are expressed as mean ± SEM (*n* = 6 mice per group; 40 fields analyzed per mouse). *p < 0.05, **p < 0.01 and ***p < 0.001.
**Figure S6:** Co‐localization analysis of TREM2 and Iba1‐positive microglia in the hippocampus and cortex of 5xFAD mice. (A) Representative confocal images showing co‐localization of TREM2 with Iba1‐positive microglia in the hippocampus and cortex across experimental groups. High‐magnification images were acquired at ×600; scale bar = 5 μm. (B) Quantification of the TREM2^+^Iba1^+^ double‐positive area in the hippocampus and cortex. (C) Quantification of the mean fluorescence intensity of TREM2^+^Iba1^+^ double‐positive signals in the hippocampus and cortex. Data are expressed as mean ± SEM (*n* = 6 mice per group; 40 fields analyzed per mouse). *p < 0.05, **p < 0.01 and ***p < 0.001.
**Figure S7:** Effects of HUCMSC transplantation via intravenous (IV) and intrathecal (IT) routes on DAP12 expression in 5xFAD mice. (A) Representative confocal images showing DAP12 immunofluorescence in the hippocampus and cortex across experimental groups. High‐magnification images were acquired at ×600; scale bar = 5 μm. (B) Quantification of DAP12‐positive area in the hippocampus and cortex. (C) Quantification of mean DAP12 fluorescence intensity in the hippocampus and cortex. Data are presented as mean ± SEM (*n* = 6 mice per group; 40 fields analyzed per mouse). *p < 0.05, **p < 0.01 and ***p < 0.001.
**Figure S8:** Co‐localization analysis of DAP12 and Iba1‐positive microglia in the hippocampus and cortex of 5xFAD mice. (A) Representative confocal images showing co‐localization of DAP12 with Iba1‐positive microglia in the hippocampus and cortex across experimental groups. High‐magnification images were acquired at ×600; scale bar = 5 μm. (B) Quantification of the DAP12^+^Iba1^+^ double‐positive area in the hippocampus and cortex. (C) Quantification of the mean fluorescence intensity of DAP12^+^Iba1^+^ double‐positive signals in the hippocampus and cortex. Data are presented as mean ± SEM (*n* = 6 mice per group; 40 fields analyzed per mouse). *p < 0.05, **p < 0.01 and ***p < 0.001.
**Figure S9:** Effects of HUCMSC transplantation via intravenous (IV) and intrathecal (IT) routes on Syk expression in 5xFAD mice. (A) Representative confocal images showing Syk immunofluorescence in the hippocampus and cortex across experimental groups. High‐magnification images were acquired at 600×; scale bar = 5 μm. (B) Quantification of Syk‐positive area in the hippocampus and cortex. (C) Quantification of mean Syk fluorescence intensity in the hippocampus and cortex. Data are shown as mean ± SEM (*n* = 6 mice per group; 40 fields analyzed per mouse). *p < 0.05, **p < 0.01 and ***p < 0.001.
**Figure S10:** Co‐localization analysis of Syk and Iba1‐positive microglia in the hippocampus and cortex of 5xFAD mice. (A) Representative confocal images showing co‐localization of Syk with Iba1‐positive microglia in the hippocampus and cortex across experimental groups. High‐magnification images were acquired at 600×; scale bar = 5 μm. (B) Quantification of the Syk^+^Iba1^+^ double‐positive area in the hippocampus and cortex. (C) Quantification of the mean fluorescence intensity of Syk^+^Iba1^+^ double‐positive signals in the hippocampus and cortex. Data are shown as mean ± SEM (*n* = 6 mice per group; 40 fields analyzed per mouse). *p < 0.05, **p < 0.01 and ***p < 0.001.

## Data Availability

All data in this research study are available from the corresponding author upon reasonable request.
