# Supplementary material for: Intrathecal Transplantation of Human Umbilical Cord‐Derived MSCs Outperforms Intravenous Administration in Attenuating Pathology and Cognitive Deficits in Alzheimer's Disease Mice
Source: CNS Neurosci Ther. 2026 Nov 4;32(11):e71215. doi: 10.1002/cns.71215 (PMC13736151; doi:10.1002/cns.71215)
Supplement: Supplementary file 1 [file CNS-32-e71215-s001.docx]

**Fig.S1 Real-time cell analysis (RTCA) growth curve of HUCMSCs**. Dynamic changes in the Cell Index (CI) of HUCMSCs were monitored over a 96-hour culture period using the RTCA system. The curve shows the dynamic growth profile of HUCMSCs over time. Data are presented as mean ± SEM from five independent experiments (*n* = 5).

**Fig.S2** **Sex-stratified analysis of Morris water maze performance in 5xFAD mice.**

(A–C) Morris water maze performance in male mice. (D–F) Morris water maze performance in female mice. (A and D) Escape latency during the 5-day training phase. (B and E) Total swimming distance during the training phase. (C and F) Swimming speed during the training phase. (G) Time spent in the target quadrant during the probe trial. (H) Distance traveled in the target quadrant during the probe trial. Data are expressed as mean ± SEM (*n* = 6 mice per group). ^*^*P* <0.05, ^**^*P* <0.01 and ^***^*P* <0.001.

**Fig. S3 Sex-stratified analysis of amyloid-β plaque burden in the hippocampus and cortex of 5xFAD mice.**

(A and C) Quantification of amyloid-β plaque burden in the hippocampus. (B and D) Quantification of amyloid-β plaque burden in the cortex. (A and B) Number of Thioflavin-S (ThS)-positive amyloid plaques per high-power field. (C and D) Average size of ThS-positive amyloid plaques in the indicated brain regions. Data are presented as mean ± SEM (*n* = 4 mice per group; 40 fields analyzed per mouse).  ^*^*P* <0.05, ^**^*P* <0.01 and ^***^*P* <0.001.

**Fig. S4 Bioinformatic analysis of differentially expressed genes (DEGs) and related pathways.** (A) Volcano plot showing DEGs. The top 15 upregulated genes are labeled in red. Dashed lines indicate the significance thresholds: |log₂FC| > 1 and adjusted P < 0.05. (B) Hierarchical clustering heatmap of the top 50 DEGs. Red indicates upregulated genes, and blue indicates downregulated genes. (C) Gene Ontology (GO) enrichment analysis of the top 10 biological processes associated with DEGs. (D) GO enrichment analysis showing representative biological processes related to microglial activation, phagocytosis, and neuroinflammatory signaling, together with associated DEGs. (E) WikiPathways enrichment analysis showing representative pathways associated with DEGs. (F) Identification of key genes, including TREM2 and TYROBP/DAP12, based on their enrichment across biological processes and pathways.

**Fig. S5 Effects of HUCMSC transplantation via intravenous (IV) and intrathecal (IT) routes on TREM2 expression in 5xFAD mice.** (A) Representative confocal images showing TREM2 immunofluorescence in the hippocampus and cortex across experimental groups. High-magnification images were acquired at ×600; scale bar = 5 μm. (B) Quantification of TREM2-positive area around amyloid plaques. (C) Quantification of mean TREM2 fluorescence intensity. Data are expressed as mean ± SEM (*n* = 6 mice per group; 40 fields analyzed per mouse). ^*^*P* <0.05, ^**^*P* <0.01 and ^***^*P* <0.001.

**Fig. S6 Co-localization analysis of TREM2 and Iba1-positive microglia in the hippocampus and cortex of 5xFAD mice.** (A) Representative confocal images showing co-localization of TREM2 with Iba1-positive microglia in the hippocampus and cortex across experimental groups. High-magnification images were acquired at ×600; scale bar = 5 μm. (B) Quantification of the TREM2⁺Iba1⁺ double-positive area in the hippocampus and cortex. (C) Quantification of the mean fluorescence intensity of TREM2⁺Iba1⁺ double-positive signals in the hippocampus and cortex. Data are expressed as mean ± SEM (*n* = 6 mice per group; 40 fields analyzed per mouse). ^*^*P* <0.05, ^**^*P* <0.01 and ^***^*P* <0.001.

**Fig. S7 Effects of HUCMSC transplantation via intravenous (IV) and intrathecal (IT) routes on DAP12 expression in 5xFAD mice.** (A) Representative confocal images showing DAP12 immunofluorescence in the hippocampus and cortex across experimental groups. High-magnification images were acquired at ×600; scale bar = 5 μm. (B) Quantification of DAP12-positive area in the hippocampus and cortex. (C) Quantification of mean DAP12 fluorescence intensity in the hippocampus and cortex. Data are presented as mean ± SEM (*n* = 6 mice per group; 40 fields analyzed per mouse). ^*^*P* <0.05, ^**^*P* <0.01 and ^***^*P* <0.001.

**Fig. S8 Co-localization analysis of DAP12 and Iba1-positive microglia in the hippocampus and cortex of 5xFAD mice.** (A) Representative confocal images showing co-localization of DAP12 with Iba1-positive microglia in the hippocampus and cortex across experimental groups. High-magnification images were acquired at ×600; scale bar = 5 μm. (B) Quantification of the DAP12⁺Iba1⁺ double-positive area in the hippocampus and cortex. (C) Quantification of the mean fluorescence intensity of DAP12⁺Iba1⁺ double-positive signals in the hippocampus and cortex. Data are presented as mean ± SEM (*n* = 6 mice per group; 40 fields analyzed per mouse). ^*^*P* <0.05, ^**^*P* <0.01 and ^***^*P* <0.001.

**Fig. S9 Effects of HUCMSC transplantation via intravenous (IV) and intrathecal (IT) routes on Syk expression in 5xFAD mice.** (A) Representative confocal images showing Syk immunofluorescence in the hippocampus and cortex across experimental groups. High-magnification images were acquired at ×600; scale bar = 5 μm. (B) Quantification of Syk-positive area in the hippocampus and cortex. (C) Quantification of mean Syk fluorescence intensity in the hippocampus and cortex. Data are shown as mean ± SEM (*n* = 6 mice per group; 40 fields analyzed per mouse). ^*^*P* <0.05, ^**^*P* <0.01 and ^***^*P* <0.001.

**Fig. S10 Co-localization analysis of Syk and Iba1-positive microglia in the hippocampus and cortex of 5xFAD mice.** (A) Representative confocal images showing co-localization of Syk with Iba1-positive microglia in the hippocampus and cortex across experimental groups. High-magnification images were acquired at ×600; scale bar = 5 μm. (B) Quantification of the Syk⁺Iba1⁺ double-positive area in the hippocampus and cortex. (C) Quantification of the mean fluorescence intensity of Syk⁺Iba1⁺ double-positive signals in the hippocampus and cortex. Data are shown as mean ± SEM (*n* = 6 mice per group; 40 fields analyzed per mouse). ^*^*P* <0.05, ^**^*P* <0.01 and ^***^*P* <0.001.
